# Choline: Exploring the Growing Science on Its Benefits for Moms and Babies

**DOI:** 10.3390/nu11081823

**Published:** 2019-08-07

**Authors:** Hunter W. Korsmo, Xinyin Jiang, Marie A. Caudill

**Affiliations:** 1 Program in Biochemistry, Graduate Center of the City University of New York, New York, NY 10016, USA; 2 Department of Health and Nutrition Sciences, Brooklyn College of the City University of New York, Brooklyn, NY 11210, USA; 3 Division of Nutritional Sciences, Cornell University, Ithaca, NY 14853, USA

**Keywords:** choline, placenta, epigenetic programming, pregnancy outcomes, cognitive development

## Abstract

The importance of ensuring adequate choline intakes during pregnancy is increasingly recognized. Choline is critical for a number of physiological processes during the prenatal period with roles in membrane biosynthesis and tissue expansion, neurotransmission and brain development, and methyl group donation and gene expression. Studies in animals and humans have shown that supplementing the maternal diet with additional choline improves several pregnancy outcomes and protects against certain neural and metabolic insults. Most pregnant women in the U.S. are not achieving choline intake recommendations of 450 mg/day and would likely benefit from boosting their choline intakes through dietary and/or supplemental approaches.

## 1. Introduction

The importance of ensuring adequate choline intakes during pregnancy is increasingly recognized. The American Medical Association (AMA) in 2017 published new advice stating that prenatal vitamin supplements should contain ‘evidence-based’ amounts of choline [1]. Similarly, in 2018, the American Academy of Pediatrics recognized choline as a ‘brain-building’ nutrient and called upon pediatricians to ensure pregnant women and young children have adequate intakes of choline [2].

Choline, like vitamin D and docosahexaenoic acid (DHA), can be synthesized in the body but not in amounts sufficient to meet metabolic demands. In 1998, choline was recognized as an essential nutrient by the Institute of Medicine (now National Academy of Medicine) when it established dietary recommendations in the form of adequate intakes [3]. The choline adequate intake (AI) level is 425 mg choline/day for women of reproductive age with upward adjustments to 450 mg choline/day during pregnancy and 550 mg choline/day during lactation [3]. Although widely distributed in the diet, choline is absent from most prenatal vitamins currently on the market, and less than ten percent of pregnant women achieve target intake levels [4].

## 2. Food Sources of Choline

Choline is found in both animal and plant source foods; however, animal source foods typically contain more choline per gram of food product. Beef, eggs, chicken, fish, and pork are concentrated sources of choline providing more than 60 mg per 100 g [5]. Among plant source foods, nuts, legumes, and cruciferous vegetables (e.g., broccoli) are relatively good sources providing at least 25 mg per 100 g [5]. Although not a concentrated source, cow’s milk is a main contributor to dietary choline intake in the U.S. [6].

Various forms of choline are found in food. The most prominent dietary form of choline is usually phosphatidylcholine (PC) with smaller amounts of free choline, phosphocholine, sphingomyelin, glycerophosphocholine, and lysophosphatidylcholine (LPC) [5]. All of these choline forms are interchangeable within the body and contribute to an individual’s “total” choline intake. Although betaine is a choline derivative, it is not considered a dietary source of choline because there is no direct route for converting betaine to choline. However, dietary betaine may have a choline-sparing effect since it participates in the remethylation of homocysteine, thus reducing the need for endogenous choline oxidation to betaine [7] and lowering the dietary requirement for choline. Upon absorption of choline, water-soluble biomolecules (free choline, phosphocholine and glycerophosphocholine) enter portal blood, while lipid-soluble forms (PC, LPC and sphingomyelin) are incorporated into chylomicrons [8].

## 3. Choline Function

**Figure 1:**
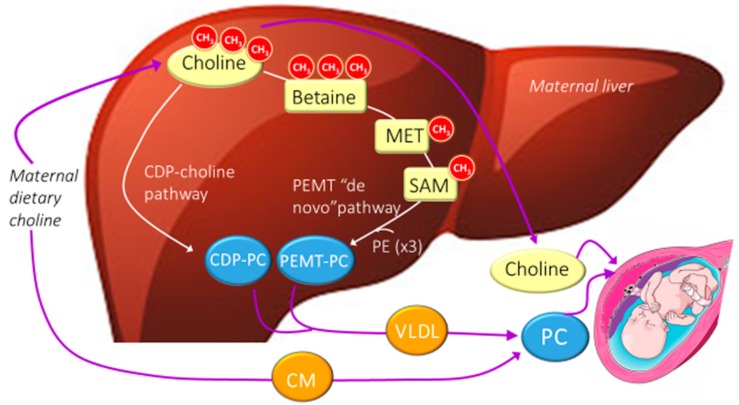
A simplified diagram of the metabolic fate of maternal dietary choline and its delivery to the developing fetus. In the liver, choline can be used to make phosphatidylcholine (PC) through the cytidine diphosphate (CDP)-choline pathway (CDP-PC), or it can be oxidized to betaine and serve as a source of methyl groups for PC synthesis via the de novo phosphatidylethanolamine *N*-methyltransferase (PEMT) pathway (PEMT-PC). Both pathways are upregulated during the third trimester of pregnancy, but PEMT-derived PC is preferentially partitioned to the developing fetus. CM, chylomicron; MET, methionine; PE, phosphatidylethanolamine; SAM, S-adenosylmethionine; VLDL, very low-density lipoprotein.

Within the body, choline is critical for a number of functions with wide-ranging roles in metabolic and physiologic processes. The choline derivative, PC, is a major constituent of all cell membranes and is required for the biosynthesis of lipoproteins, including very low-density lipoproteins (VLDLs), which facilitate the hepatic export of lipid (Figure 1). Inadequate choline intake disturbs the integrity of cellular membranes resulting in ‘leaky membranes’ [9] and impairs the mobilization of fat from liver resulting in fatty liver [10]. Acetylcholine functions as a neurotransmitter in both the central nervous system (CNS) and the peripheral nervous system (PNS). In the CNS, cholinergic projections from the basal forebrain to the cerebral cortex and hippocampus support the cognitive functions of those target areas [11]. In the PNS, acetylcholine activates skeletal muscle and is a major neurotransmitter in the autonomic nervous system [12]. Sphingomyelin is a constituent of the myelin sheath [13], which covers the axons of nerve cells and facilitates efficient transmission of nerve signals. Finally, betaine is a source of methyl groups for *S*-adenosylmethionine (SAM)-dependent methyltransferases and for folate mediated one-carbon metabolism following the demethylation of dimethylglycine (produced when betaine is used as a methyl donor) within the mitochondria of hepatocytes [8].

## 4. Choline Metabolism.

Choline metabolism to its various biomolecules occurs mostly in the liver (Figure 1), with the exception of acetylcholine generation, which occurs in both cholinergic neurons and the placenta [14]. In liver (and other nucleated cells), free choline is initially partitioned to the cytidine diphosphate (CDP)-choline pathway (also known as the Kennedy pathway) for the generation of PC, a phospholipid that can further contribute its phosphocholine headgroup to ceramide for the synthesis of sphingomyelin. Alternatively, free choline can be oxidized to betaine in a two-step irreversible reaction catalyzed by choline dehydrogenase (CHDH) and betaine aldehyde dehydrogenase. The oxidation of choline to betaine has been referred to as a ‘spillover’ pathway [15] that is most active under conditions of surplus choline. Betaine participates in the remethylation of homocysteine to methionine by betaine-homocysteine *S*-methyltransferase (BHMT). Methionine can subsequently be converted to SAM, the universal methyl donor for over 100 cellular methylation reactions including the phosphatidylethanolamine *N*-methyltransferase (PEMT) pathway.

The PEMT pathway facilitates de novo choline biosynthesis whereby phosphatidylethanolamine (PE), a non-choline containing phospholipid, undergoes sequential methylation to PC using SAM as the methyl donor (Figure 1). Primary sources of the methyl groups used for SAM-dependent methylation of PE include methionine, methyl-folate, and choline itself following its oxidation to betaine. Indeed, tracer studies in pregnant women have demonstrated substantial use of choline-derived methyl groups for the synthesis of PC via the PEMT pathway [16]. An explanation for this apparent inefficiency may stem from differences in the fatty acid composition of the PC molecules derived from the PEMT pathway as opposed to the CDP-choline pathway. PEMT derived PC molecules are enriched in long-chain polyunsaturated fatty acids (PUFAs) (C18–C22), whereas CDP-PC molecules are enriched in saturated fatty acids (C16, C18) [17]. Both PEMT and CDP-choline derived PC molecules can be incorporated into VLDL and exported from liver to circulation for uptake by other tissues including the placenta (Figure 1). 

During the third trimester of pregnancy, the activities of the CDP-choline and PEMT pathways are upregulated; however, only the PC products derived from the PEMT pathway are enriched in cord plasma [16], demonstrating preferential transport of PEMT derived PC to the developing fetus. This preferential shuttling of PEMT-derived PCs during the third trimester may be due to its enrichment in DHA (22:6n3), a polyunsaturated omega 3 fatty acid that accumulates in the neonatal brain during the third trimester of pregnancy [18]. Notably, studies in women of reproductive age have shown that choline supplementation upregulates the PEMT pathway [16] and results in greater enrichment of PC-DHA in circulating red blood cells [19], suggesting that choline supplementation may be a strategy for improving the bioavailability of DHA. 

In addition to PC-DHA, LPC-DHA, a hydrolytic product of PEMT-PC, may represent another source of DHA for the developing fetus [20]. LPC-DHA circulates bound to albumin and can be taken up by the placenta via the major facilitator superfamily domain 2 protein (MFSD2A), an LPC symporter with high affinity for LPC-DHA [21,22]. Genetic loss of MFSD2A expression in mice results in impaired accretion of DHA in the fetal brain and eye, suggesting that LPC-DHA is a critical form of DHA for transport across the placenta, the fetal blood-brain barrier and/or the retinal epithelium [23].

The metabolism of choline is influenced by single nucleotide polymorphisms (SNPs) found in genes that encode enzymes in folate and choline dependent pathways [24]. For example, loss-of-function variants in folate metabolizing enzymes (e.g., methylenetetrahydrofolate reductase [MTHFR], 5-methyltetrahydrofolate-homocysteine methyltransferase [MTR], and methylenetetrahydrofolate dehydrogenase 1 [MTHFD1]) strain cellular PC production, possibly via impaired folate-dependent PEMT-PC biosynthesis, even when the choline AI is consumed [25]. Similarly, disturbances in the metabolic partitioning of choline have been observed at levels of choline intake approximating the choline AI among women harboring SNPs within choline metabolizing enzymes (PEMT, CHDH, BHMT, and choline kinase alpha [CHKA]) [26]. Some of these SNPs have also been shown to influence the risk of developing organ dysfunction, a manifestation of choline deficiency, when dietary choline intake is restricted [27]. Overall these “risk” genotypes would be expected to increase the dietary requirement for choline.

## 5. Choline and Fetal Development

During fetal development, large amounts of choline-derived phospholipids, such as phosphatidylcholine and sphingomyelin, are needed to support rapid cell division, growth, and myelination. The choline derived neurotransmitter acetylcholine influences many processes in the developing brain (e.g., progenitor cell proliferation and differentiation, neurogenesis, gliogenesis, cell survival, morphology and migration, and synaptic plasticity) [28,29], and supports normal development of the hippocampus [30], a region of the brain with roles in learning, memory, and attention. As a major source of methyl groups, choline also facilitates methylation of cytosine residues within promoter regions of the placental and fetal genome, an epigenetic modification that influences gene expression and can have lasting effects on metabolic and physiologic processes [31].

## 6. Choline and Pregnancy Outcomes

**Figure 2:**
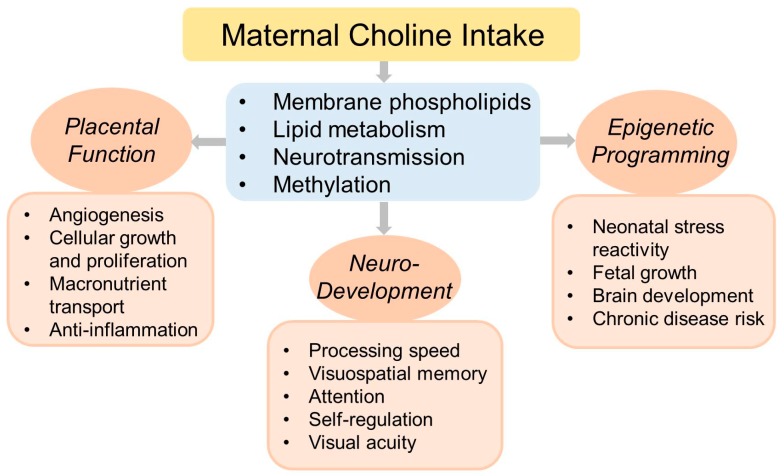
An overview of the effects of maternal choline intake during pregnancy on physiological processes and health outcomes.

Choline intake during pregnancy has been shown to influence numerous metabolic and physiologic processes as outlined below and highlighted in Figure 2.

### 6.1. Epigenetic Programming of Postnatal Health

As a methyl donor, choline influences DNA and histone methylation – two central epigenomic processes that regulate gene expression [31]. During murine pregnancy, maternal choline deficiency has been shown to modulate the epigenome of the offspring, with lasting adverse effects on functions related to fetal growth [32] as well as brain development and angiogenesis [33,34]. Rodent studies have also demonstrated modulatory effects of maternal choline supplementation during pregnancy on the placental and fetal epigenome [35,36,37], with some reports of reduced disease risk [36,37]. In humans, gestational choline may influence offspring stress reactivity. Consumption of a higher maternal choline intake (930 vs. 480 mg choline/day) throughout the third trimester of pregnancy increased promoter region methylation of the placental corticotropin-releasing hormone (CRH) gene and decreased its expression (Table 1) [38]. CRH is a regulatory protein in the hypothalamus-pituitary-adrenal (HPA) axis that mediates stress reactivity by stimulating cortisol production by the adrenal gland. Like the brain, the placenta makes CRH, which can subsequently enter fetal circulation and activate the HPA axis [39]. As expected, this same study found lower cord blood cortisol concentrations in the newborns of mothers consuming 930 vs. 480 mg choline/day. Because a heightened response to stress increases the risk of depression, hypertension, type 2 diabetes mellitus, and immunological disorders later in life [40,41], the children with decreased stress-reactivity at birth may be less likely to develop mental and cardio-metabolic diseases [42]. 

### 6.2. Placental Function

The placenta is a critical organ of pregnancy that mediates nutrient and oxygen supply to the developing fetus. Proper functioning of the placenta depends on development of a vasculature that enables sufficient blood flow to the developing fetus. Inadequate vascularization of the placenta can lead to pregnancy disorders characterized by impaired fetal growth such as intrauterine growth restriction (IUGR) and preeclampsia [57]. Emerging data from a growing number of studies suggest that choline supply can beneficially influence functional processes of the placenta, including angiogenesis [50,58,59], inflammation [58,59,60], and macronutrient transport [61]. Based on rodent data, prenatal choline may also be a nutritional approach to mitigate placental insufficiency [60,62]. In the Dlx3 ± mouse model of placental insufficiency, gestational choline improved early fetal growth [62], possibly by increasing the size of the placental labyrinth [60], a region of the placenta that contains the villi where nutrients pass from the maternal blood into the fetal blood. 

In humans, consumption of additional choline (930 vs. 480 mg choline/day) during the third trimester of pregnancy reduced the production of placental soluble fms-like tyrosine kinase 1 (sFlt1) [50] (Table 1), an anti-angiogenic protein that sequesters vascular endothelial growth factor (VEGF) in maternal circulation and contributes to endothelial dysfunction, hypertension, and proteinuria in preeclampsia. Downregulation of placental sFLT1 production by high choline was also demonstrated in an in-vitro follow-up study using human trophoblast cells [58], an effect that appeared to be mediated through attenuation of the protein kinase C (PKC) signaling pathway. 

### 6.3. Macronutrient Metabolism and Energy Homeostasis

Several studies have demonstrated an interaction between choline and macronutrient metabolism during pregnancy [63,64,65]. In a mouse model of high-fat feeding-induced gestational diabetes mellitus (GDM), Nam et al. [63] found that maternal choline supplementation prevented fetal overgrowth during mid-gestation, the most common complication of GDM. This effect was associated with a dampening of the mechanistic target of rapamycin (mTOR) signaling pathway in the placenta, which promotes placental transport of glucose and fat [63,66,67]. A follow-up study at a later gestational time point reported that maternal choline supplementation normalized whole-body adiposity and reduced hepatic triglyceride accumulation possibly by downregulating lipogenic gene expression in the GDM mouse embryos [64]. In addition, an examination of the placental structure suggested that choline supplementation mitigated the increase in both placental junctional zone thickness and number of glycogen cells in GDM rodent placentas [65]. Betaine supplementation of GDM mice showed similar, but not identical, phenotypic outcomes suggesting that the beneficial effects of choline supplementation on GDM fetal metabolism may be partially mediated via its oxidation to betaine [68]. While the long-term outcome of maternal choline supplementation on GDM offspring metabolism remains to be determined, methyl donor supplementation during murine pregnancy has been shown to block some of the adverse effects of maternal high fat feeding on offspring physiology possibly by reversing diet-induced global hypomethylation within the offspring CNS [69].

### 6.4. Neurodevelopment and Cognitive Function

Studies in rodents have consistently shown that high choline intake during gestation improves cognitive function in adulthood and prevents the memory decline associated with old age [70,71,72]. Likewise, many studies have shown that choline inadequacy during gestation adversely influences offspring brain development and function [33,34,73,74,75,76]. For example, piglets born to choline-deficient mothers had lower brain weight, volume, and less white and grey matter at 30 days of age as compared to control piglets [73,74,75]. A recent study reported that low choline during gestation also disrupted retinal development and visual function in mice [77]. Possible mechanisms by which maternal choline influences offspring neurodevelopment and cognitive function are related to (i) the use of choline for phospholipid membrane synthesis; (ii) facilitation of DHA uptake [21]; (iii) myelination of neurons during early development [13,78]; (iv) alterations in hippocampal acetylcholine metabolism [28,79]; (v) modulation of neurogenesis and neuronal differentiation [76,77]; and (vi) modifications on epigenetic marks that govern hippocampal angiogenesis and cellular proliferation [33,34]. All of these choline-induced outcomes ultimately influence cellular proliferation, differentiation, morphology, dendritic branching, neurogenesis, and potentiation of the offspring hippocampus [76,80,81,82].

A few prospective observational studies have explored the relationship between maternal choline status (intake or blood levels) during human pregnancy and cognitive development in children (Table 1). Wu et al. found that concentrations of maternal plasma choline and betaine at 16 weeks of gestation were positively associated with infant cognitive test scores at 18 months [46]. The Project Viva study observed an association of better visual memory among 7-year-old children of mothers with choline intakes in the top versus the bottom quartile during the second trimester of pregnancy [49]. Most recently, higher serum choline concentrations in mothers with infections at gestational week 16 were associated with better inhibition of auditory cerebral response in newborns and improved development of self-regulation in 1-year old infants [55].

Findings from randomized clinical trials (RCTs) in support of a beneficial effect of prenatal choline on cognitive outcomes are also beginning to emerge (Table 1). In a randomized controlled feeding study, faster processing speed was observed among infants born to mothers consuming 930 versus 480 mg choline/day during their third trimester of pregnancy [53]. Moreover, children whose mothers consumed 930 (versus 480) mg choline/day performed significantly better on a task of color-location memory at age 7 years, suggesting a long-term beneficial effect of prenatal choline supplementation on offspring cognition [56]. Another RCT reported improved cerebral inhibition, an indicator of attention, among offspring at 5 weeks of age [51], and reduced attentional problems and social withdrawal at 40 months of age [52] with choline supplementation given from the second trimester of pregnancy, and then postnatally to the infant.

However, not all studies have found a relationship between maternal choline status/intake and indicators of offspring cognition, which may indicate (among other things) that not all aspects of cognition are choline-responsive. Signore et al. [44] found no associations between cord blood choline concentrations and intellectual quotient (IQ) scores in children at 5 years of age, while Villamor et al. [47] reported no associations between maternal choline intake and vocabulary or visual-motor scores in children at 3 years of age. In addition, an RCT [48] that randomized women to supplemental choline (750 mg/day) or placebo from 18 weeks gestation through 90 days postpartum reported no benefit of maternal choline supplementation on infant cognition as assessed using a variety of cognitive domains including general cognitive function, language development, and episodic and visuospatial memory.

### 6.5. Protection from Neural Insults

In addition to improving some aspects of offspring cognition, findings from animal studies show that perinatal choline protects the brain from the neuropathological changes associated with Alzheimer’s disease (AD) [83,84], fetal alcohol syndrome [85,86,87], autism [88,89], Down syndrome [90,91,92,93], and early-life iron deficiency [94,95]. In the Ts65DN mouse model of Down syndrome, maternal choline supplementation improved spatial memory and hippocampal neurogenesis in the brains of the Ts65Dn offspring [90]. Notably, mechanistic studies in which a choline tracer was administered to the adult Ts65DN offspring indicated that prenatal choline permanently upregulated PEMT activity and the delivery of PEMT derived PC (enriched in DHA) to the brains of these mice [93]. Maternal choline supplementation also normalized the number and density of cholinergic neurons in the medial septum [92]. Since the loss of cholinergic neurons is often seen in AD, studies on the trisomy mice highlight the potential of maternal choline supplementation as a preventative measure for early-onset AD [96]. Indeed, in the APP/PS1 mouse model of AD, perinatal choline supplementation reduced the number and total area of amyloid plaque [83]. Interestingly, cross-generational effects of perinatal choline have been observed with fewer cognitive deficits in the F2 generation of APP/PS1 mice born to F1 mice that were not supplemented with additional choline during pregnancy themselves [84]. Prenatal choline supplementation also mitigated long-term neurobehavioral abnormalities and transcriptomic alterations in gene networks associated with autism and schizophrenia in rat offspring exposed to fetal-neonatal iron deficiency [94,95].

Although few human studies have explored the effects of maternal choline supplementation on cognitive deficits arising from neural insults, preliminary data from a recent study found that this intervention mitigated some of the adverse effects of prenatal alcohol exposure on infant growth and cognitive function (Table 1) [54]. These beneficial effects of prenatal choline in fetal alcohol exposure echoed the data from alcohol-exposed animals and could be related to choline’s role as a methyl donor and its ability to counter epigenetic changes that occur in various brain regions as a consequence of prenatal alcohol exposure [97,98]. Prenatal choline may also protect against the development of congenital malformations of the central nervous system, commonly referred to as neural tube defects (NTDs). For example, both maternal choline intake and biomarkers of choline status during pregnancy have been inversely associated with offspring NTD risk [43,45].

## 7. Choline Requirements during Pregnancy

The endogenous synthesis of choline via the PEMT pathway is elevated during the second half of pregnancy due to increases in estrogen and upregulation of the PEMT gene, which contains estrogen response elements within its promoter region [99]. However, as previously mentioned, the upregulation of the PEMT pathway increases the demand for methyl groups from various sources including choline itself [16]. Indeed, data from human feeding studies suggest that the choline adequate intake (AI) of 450 mg/day may not be sufficient in meeting the demands of pregnancy. For example, pregnant women consuming 480 mg choline/day exhibit 40–60% lower circulating concentrations of the choline-derived methyl metabolites, betaine, dimethylglycine, and sarcosine as compared to nonpregnant women consuming this same level of choline intake [100]. The partitioning of choline towards betaine (versus the CDP-choline pathway) is also diminished among pregnant (versus nonpregnant) women with choline intakes of 480 mg/day, despite greater use of betaine as a methyl donor in the pregnant state [16]. Notably, a doubling of choline intake during pregnancy (i.e., 930 versus 480 mg choline/day) restores the partitioning of choline between the oxidative and CDP-choline pathways to that of a nonpregnant state [16]; it also increases circulating concentrations of choline-derived methyl metabolites [100], increases placental DNA methylation [38], and overcomes some of the metabolic inefficiencies caused by common polymorphisms in folate and choline metabolizing genes [25,26]. Moreover, this higher level of choline intake beneficially influenced several pregnancy outcomes including improvements in indicators of neonatal stress reactivity [38], reductions in placental sFLT1 production [50], faster information processing speed among infants [53], and better memory in children at age 7 years [56].

## 8. Safety

The tolerable upper intake level (UL) of choline is 3.5 g/day for adults and was established to prevent hypotension and fishy body odor [3]. To date, none of the RCTs conducted in healthy pregnant women have reported any adverse effects of choline supplementation at levels ranging from 550–900 mg/day [48,51,100]. However, a few animal studies have reported that supplementing the maternal diet with very high levels of methyl nutrients (with choline as one of the components) increased susceptibility to colitis [101] and asthma-like allergic airway disease [102], as well as impaired energy homeostasis when combined with prenatal protein restriction [103]. In addition, choline can be converted to trimethylamine *N*-oxide (TMAO), a risk indicator of cardio-metabolic diseases, via the action of gut microbes. However, whether TMAO has a causative role in human chronic disease development is unknown [104,105], and the long-term influence of choline intake on maternal and offspring TMAO status and health requires further investigation. In summary, certain aspects of fetal development may follow a U-shaped response pattern to choline supply (and other methyl donors), whereby both low and excessively high exposures could adversely influence offspring health.

## 9. Conclusions and Future Directions

Data from both animal and human studies highlight the importance of ensuring an adequate choline intake during pregnancy. Supplementing the maternal diet with additional choline has been shown to improve offspring cognition, neurodevelopment, and placental functioning, and to protect against neural and metabolic insults. However, RCTs on prenatal choline and pregnancy outcomes are limited and the dose–response relationship between maternal choline intake and offspring health remains to be fully discerned. Future clinical interventions with larger sample sizes, multiple dosages, specific endpoints with clinical utility, mechanistic measurements, and long-term follow-up are needed to establish maternal choline intake recommendations that optimize fetal development and reduce the risk of pregnancy complications. In the interim, consumption of 450–1000 mg choline/day appears to be an intake level that would support fetal development and improve pregnancy outcomes based on the available and emerging science.

## Figures and Tables

**Table 1:** Human studies with a focus on maternal choline intake (or status) and pregnancy and child health outcomes (in chronological order).

Study	Design	Intervention/Choline Marker Measurement	Pregnancy and Child Health Outcomes	References
Shaw et al. 2004	Case-control	Maternal dietary choline intake during the 3 months before conception	Reduced neural tube defect (NTD) risk with higher maternal choline intakes (*n* = 424 NTD and 440 control)	[43]
Signore et al. 2008	Prospective cohort	Maternal serum total and free choline throughout gestation and cord blood choline concentrations	No association between child intelligence quotient (IQ) scores at 5 years of age and maternal or cord blood choline (*n* = 404 maternal-child pairs)	[44]
Shaw et al. 2009	Prospective case-control	Serum total choline concentrations during the gestational week 15–18	Reduced NTD risk with higher serum choline concentrations (*n* = 80 NTD and 409 control)	[45]
Wu et al. 2012	Prospective cohort	Maternal plasma free choline at gestational week 16	Better cognitive scores in 18-month-old infants with higher maternal plasma free choline levels (*n* = 154 maternal-child pairs)	[46]
Villamor et al. 2012	Prospective cohort	Maternal dietary choline intake in the 1st and 2nd trimester of pregnancy	No association between cognitive performance in 3-year-old children and maternal choline intake (*n* = 1210)	[47]
Jiang et al. 2012 ^a^	Randomized clinical trial (RCT)	Controlled feeding of 930 versus 480 mg choline/day for 12 weeks during 3rd trimester of pregnancy	Higher placental CRH promoter methylation and lower cord blood cortisol concentrations in the 930 mg/d (*n* = 13) versus 480 mg/day (*n* = 13) choline intake group.	[38]
Cheatham et al. 2012	RCT	Phosphatidylcholine (PC) supplement (750 mg choline/day) from 2nd trimester of pregnancy to 90 days postpartum	No effect on development or memory in 10 or 12-month-old infants (intervention group *n* = 49, placebo group *n* = 50)	[48]
Boeke et al. 2013	Prospective cohort	Maternal dietary choline intake in the 2nd trimester of pregnancy	Better visual memory in 7-year-old children with top interquartile dietary choline intake during pregnancy (*n* = 895)	[49]
Jiang et al. 2013 ^a^	RCT	Controlled feeding of 930 versus 480 mg choline/day for 12 weeks during 3rd trimester of pregnancy	Lower placental sFlt1 mRNA expression and maternal serum sFLT1 levels in the 930 mg/day (*n* = 13) versus 480 mg/d (*n* = 13) choline intake group	[50]
Ross et al. 2013 ^b^	RCT	PC supplement (900 mg choline/day) from 2nd trimester of pregnancy until delivery; 100mg/day of PC to infants until 3 months of age	Greater attention development in 5-week-old infants in the intervention group (*n* = 36) versus control (*n* = 40)	[51]
Ross et al. 2016 ^b^	RCT	PC supplement (900 mg choline/day) from 2nd trimester of pregnancy until delivery; 100mg/day of PC to infants until 3 months of age	Reduced attentional problems and social withdrawal in children at 40 months of age in the intervention group (*n* = 23) versus control (*n* = 26)	[52]
Caudill et al. 2018 ^a^	RCT	Controlled feeding of 930 versus 480 mg choline/day for 12 weeks during 3rd trimester of pregnancy	Faster information processing speed in infants during 4–13 months in the 930 (*n* = 12) versus 480 mg/day group (*n* = 12)	[53]
Jacobson et al. 2018	RCT	2 g choline/day or placebo from mid-pregnancy until delivery among heavy alcohol drinkers	Better eyeblink conditioning in infants at 6.5 months, higher novelty preference scores at 12 months, and more catch-up growth at both time points in the choline treated group (*n* = 32) versus control (*n* = 31)	[54]
Freedman et al. 2018	Prospective cohort	Serum-free choline and betaine concentrations at week 16 of gestation	Improved development of cerebral inhibition in newborns and behavioral regulation in 1-year-old infants born to infected mothers (*n* = 66) with higher gestational serum choline concentrations.	[55]
Bahnfleth et al. 2019 ^a^	RCT	Controlled feeding of 930 versus 480 mg choline/day for 12 weeks during 3rd trimester of pregnancy	Better performance on a task of color-location memory at age 7 years in the 930 mg choline/day (*n* = 11) versus 480 choline mg/day (*n* = 9) choline intake group.	[56]

^a^ These articles are based on the same controlled feeding study; ^b^ These articles are based on the same RCT.

## References

[1] AMA Wire AMA Backs Global Health Experts in Calling Infertility a Disease. https://wire.ama-assn.org/ama-news/ama-backs-global-health-experts-calling-infertility-disease.

[2] Schwarzenberg S.J., Georgieff M.K., Committee on Nutrition (2018). Advocacy for Improving Nutrition in the First 1000 Days to Support Childhood Development and Adult Health. Pediatrics.

[3] Institute of Medicine (1998). Dietary Reference Intakes for Thiamin, Riboflavin, Niacin, Vitamin B6, Folate, Vitamin B12, Pantothenic Acid, Biotin and Choline.

[4] Wallace T.C., Fulgoni V.L. (2017). Usual Choline Intakes Are Associated with Egg and Protein Food Consumption in the United States. Nutrients.

[5] Patterson Y., Bhagwat A., Williams R., Howe C., Holden M. (2008). USDA Database for The Choline Content of Common Foods.

[6] Chester D., Goldman J., Ahuja J., Moshfegh A. Dietary Intakes of Choline: What We Eat in America, NHANES 2007-Food Surveys Research Group Dietary Data Brief No. October 2011. http://ars.usda.gov/Services/docs.htm?docid=19476.

[7] Dilger R.N., Garrow T.A., Baker D.H. (2007). Betaine can partially spare choline in chicks but only when added to diets containing a minimal level of choline. J. Nutr..

[8] Klatt K., Caudill M., Stipanuk M., Caudill M. (2018). Folate, Choline, Vitamin B12 and Vitamin B6. Biochemical, Physiological, & Molecular Aspects of Human Nutrition.

[9] Li Z., Agellon L.B., Allen T.M., Umeda M., Jewell L., Mason A., Vance D.E. (2006). The ratio of phosphatidylcholine to phosphatidylethanolamine influences membrane integrity and steatohepatitis. Cell Metab..

[10] Zeisel S.H., Klatt K.C., Caudill M.A. (2018). Choline. Adv. Nutr..

[11] Ballinger E.C., Ananth M., Talmage D.A., Role L.W. (2016). Basal Forebrain Cholinergic Circuits and Signaling in Cognition and Cognitive Decline. Neuron.

[12] McCorry L.K. (2007). Physiology of the Autonomic Nervous System. Am. J. Pharm. Educ..

[13] Morell P., Quarles R., Siegel G.J., Agranoff B.W., Albers R.W., Fisher S.K., Uhler M.D. (1999). Characteristic Composition of Myelin. Basic Neurochemistry: Molecular, Cellular and Medical Aspects.

[14] Kwan S., King J., Caudill M., Duttaroy A., Basak S. (2015). Choline and Placental Trophoblast Development. Human Placental Trophoblasts.

[15] Park E.I., Garrow T.A. (1999). Interaction between Dietary Methionine and Methyl Donor Intake on Rat Liver Betaine-homocysteine Methyltransferase Gene Expression and Organization of the Human Gene. J. Biol. Chem..

[16] Yan J., Jiang X., West A.A., Perry C.A., Malysheva O.V., Brenna J.T., Stabler S.P., Allen R.H., Gregory J.F., Caudill M.A. (2013). Pregnancy alters choline dynamics: Results of a randomized trial using stable isotope methodology in pregnant and nonpregnant women. Am. J. Clin. Nutr..

[17] Delong C.J., Shen Y.-J., Thomas M.J., Cui Z. (1999). Molecular Distinction of Phosphatidylcholine Synthesis between the CDP-Choline Pathway and Phosphatidylethanolamine Methylation Pathway. J. Biol. Chem..

[18] Clandinin M., Chappell J., Leong S., Heim T., Swyer P., Chance G. (1980). Intrauterine fatty acid accretion rates in human brain: Implications for fatty acid requirements. Early Hum. Dev..

[19] A West A., Yan J., Jiang X., A Perry C., Innis S.M., A Caudill M. (2013). Choline intake influences phosphatidylcholine DHA enrichment in nonpregnant women but not in pregnant women in the third trimester. Am. J. Clin. Nutr..

[20] Lagarde M., Bernoud N., Brossard N., Lemaitre-Delaunay D., Thies F., Croset M., Lecerf J. (2001). Lysophosphatidylcholine as a Preferred Carrier Form of Docosahexaenoic Acid to the Brain. J. Mol. Neurosci..

[21] Nguyen L.N., Ma D., Shui G., Wong P., Cazenave-Gassiot A., Zhang X., Wenk M.R., Goh E.L.K., Silver D.L. (2014). Mfsd2a is a transporter for the essential omega-3 fatty acid docosahexaenoic acid. Nature.

[22] Ferchaud-Roucher V., Kramer A., Silva E., Pantham P., Weintraub S.T., Jansson T., Powell T.L. (2019). A potential role for lysophosphatidylcholine in the delivery of long chain polyunsaturated fatty acids to the fetal circulation. Biochim. Biophys. Acta Mol. Cell Biol. Lipids.

[23] Wong B.H., Chan J.P., Cazenave-Gassiot A., Poh R.W., Foo J.C., Galam D.L., Ghosh S., Nguyen L.N., Barathi V.A., Yeo S.W. (2016). Mfsd2a Is a Transporter for the Essential ω-3 Fatty Acid Docosahexaenoic Acid (DHA) in Eye and Is Important for Photoreceptor Cell Development. J. Biol. Chem..

[24] Ganz A.B., Klatt K.C., Caudill M.A. (2017). Common Genetic Variants Alter Metabolism and Influence Dietary Choline Requirements. Nutrients.

[25] Ganz A.B., Shields K., Fomin V.G., Lopez Y.S., Mohan S., Lovesky J., Chuang J.C., Ganti A., Carrier B., Yan J. (2016). Genetic impairments in folate enzymes increase dependence on dietary choline for phosphatidylcholine production at the expense of betaine synthesis. FASEB J..

[26] Ganz A.B., Cohen V.V., Swersky C.C., Stover J., Vitiello G.A., Lovesky J., Chuang J.C., Shields K., Fomin V.G., Lopez Y.S. (2017). Genetic Variation in Choline-Metabolizing Enzymes Alters Choline Metabolism in Young Women Consuming Choline Intakes Meeting Current Recommendations. Int. J. Mol. Sci..

[27] Da Costa K.-A., Kozyreva O.G., Song J., Galanko J.A., Fischer L.M., Zeisel S.H. (2006). Common genetic polymorphisms affect the human requirement for the nutrient choline. FASEB J..

[28] Abreu-Villaça Y., Filgueiras C.C., Manhães A.C. (2011). Developmental aspects of the cholinergic system. Behav. Brain Res..

[29] Lauder J.M., Schambra U.B. (1999). Morphogenetic Roles of Acetylcholine. Environ. Heal. Perspect..

[30] Blusztajn J.K., Rinnofner J. (2016). Intrinsic Cholinergic Neurons in the Hippocampus: Fact or Artifact?. Front. Synaptic Neurosci..

[31] Blusztajn J.K., Mellott T.J. (2012). Choline nutrition programs brain development via DNA and histone methylation. Central Nerv. Syst. Agents Med. Chem..

[32] Kovacheva V.P., Mellott T.J., Davison J.M., Wagner N., Lopez-Coviella I., Schnitzler A.C., Blusztajn J.K. (2007). Gestational Choline Deficiency Causes Global and Igf2 Gene DNA Hypermethylation by Up-regulation ofDnmt1Expression. J. Biol. Chem..

[33] Mehedint M.G., Craciunescu C.N., Zeisel S.H. (2010). Maternal dietary choline deficiency alters angiogenesis in fetal mouse hippocampus. Proc. Natl. Acad. Sci. USA.

[34] Mehedint M.G., Niculescu M.D., Craciunescu C.N., Zeisel S.H. (2010). Choline deficiency alters global histone methylation and epigenetic marking at the Re1 site of the calbindin 1 gene. FASEB J..

[35] Kwan S.T.C., King J.H., Grenier J.K., Yan J., Jiang X., Roberson M.S., Caudill M.A. (2018). Maternal Choline Supplementation during Normal Murine Pregnancy Alters the Placental Epigenome: Results of an Exploratory Study. Nutrients.

[36] Medici V., Shibata N.M., Kharbanda K.K., Islam M.S., Keen C.L., Kim K., Tillman B., French S.W., Halsted C.H., LaSalle J.M. (2014). Maternal choline modifies fetal liver copper, gene expression, DNA methylation, and neonatal growth in the tx-j mouse model of Wilson disease. Epigenetics.

[37] Kovacheva V.P., Davison J.M., Mellott T.J., Rogers A.E., Yang S., O’Brien M.J., Blusztajn J.K. (2009). Raising gestational choline intake alters gene expression in DMBA-evoked mammary tumors and prolongs survival. FASEB J..

[38] Jiang X., Yan J., West A.A., Perry C.A., Malysheva O.V., Devapatla S., Pressman E., Vermeylen F., Caudill M.A. (2012). Maternal choline intake alters the epigenetic state of fetal cortisol-regulating genes in humans. FASEB J..

[39] Majzoub J.A., Karalis K.P. (1999). Placental corticotropin-releasing hormone: Function and regulation. Am. J. Obstet. Gynecol..

[40] Levitt N.S., Lambert E.V., Woods D., Hales C.N., Andrew R., Seckl J.R. (2000). Impaired Glucose Tolerance and Elevated Blood Pressure in Low Birth Weight, Nonobese, Young South African Adults: Early Programming of Cortisol Axis. J. Clin. Endocrinol. Metab..

[41] Xiong F., Zhang L. (2013). Role of the hypothalamic-pituitary-adrenal axis in developmental programming of health and disease. Front. Neuroendocrinol..

[42] Chen M., Zhang L. (2011). Epigenetic mechanisms in developmental programming of adult disease. Drug Discov. Today.

[43] Shaw G.M., Carmichael S.L., Yang W., Selvin S., Schaffer D.M. (2004). Periconceptional Dietary Intake of Choline and Betaine and Neural Tube Defects in Offspring. Am. J. Epidemiol..

[44] Signore C., Ueland P.M., Troendle J., Mills J.L. (2008). Choline concentrations in human maternal and cord blood and intelligence at 5 y of age. Am. J. Clin. Nutr..

[45] Shaw G.M., Finnell R.H., Blom H.J., Carmichael S.L., Vollset S.E., Yang W., Ueland P.M. (2009). Choline and Risk of Neural Tube Defects in a Folate-fortified Population. Epidemiology.

[46] Wu B.T., Dyer R.A., King D.J., Richardson K.J., Innis S.M. (2012). Early second trimester maternal plasma choline and betaine are related to measures of early cognitive development in term infants. PLoS ONE.

[47] Villamor E., Rifas-Shiman S.L., Gillman M.W., Oken E. (2012). Maternal intake of methyl-donor nutrients and child cognition at 3 years of age. Paediatr. Périnat. Epidemiol..

[48] Cheatham C.L., Goldman B.D., Fischer L.M., Da Costa K.-A., Reznick J.S., Zeisel S.H. (2012). Phosphatidylcholine supplementation in pregnant women consuming moderate-choline diets does not enhance infant cognitive function: A randomized, double-blind, placebo-controlled trial. Am. J. Clin. Nutr..

[49] Boeke C.E., Gillman M.W., Hughes M.D., Rifas-Shiman S.L., Villamor E., Oken E. (2013). Choline intake during pregnancy and child cognition at age 7 years. Am. J. Epidemiol..

[50] Jiang X., Bar H.Y., Yan J., Jones S., Brannon P.M., West A.A., Perry C.A., Ganti A., Pressman E., Devapatla S. (2013). A higher maternal choline intake among third-trimester pregnant women lowers placental and circulating concentrations of the antiangiogenic factor fms-like tyrosine kinase-1 (sFLT1). FASEB J..

[51] Ross R.G., Hunter S.K., McCarthy L., Beuler J., Hutchison A.K., Wagner B.D., Leonard S., Stevens K.E., Freedman R. (2013). Perinatal choline effects on neonatal pathophysiology related to later schizophrenia risk. Am. J. Psychiatry.

[52] Ross R.G., Hunter S.K., Hoffman M.C., McCarthy L., Chambers B.M., Law A.J., Leonard S., Zerbe G.O., Freedman R. (2016). Perinatal Phosphatidylcholine Supplementation and Early Childhood Behavior Problems: Evidence for CHRNA7 Moderation. Am. J. Psychiatry.

[53] Caudill M.A., Strupp B.J., Muscalu L., Nevins J.E.H., Canfield R.L. (2018). Maternal choline supplementation during the third trimester of pregnancy improves infant information processing speed: A randomized, double-blind, controlled feeding study. FASEB J..

[54] Jacobson S.W., Carter R.C., Molteno C.D., Stanton M.E., Herbert J.S., Lindinger N.M., Lewis C.E., Dodge N.C., Hoyme H.E., Zeisel S.H. (2018). Efficacy of Maternal Choline Supplementation During Pregnancy in Mitigating Adverse Effects of Prenatal Alcohol Exposure on Growth and Cognitive Function: A Randomized, Double-Blind, Placebo-Controlled Clinical Trial. Alcohol Clin. Exp. Res..

[55] Freedman R., Hunter S.K., Law A.J., Wagner B.D., D’Alessandro A., Christians U., Noonan K., Wyrwa A., Hoffman M.C. (2019). Higher Gestational Choline Levels in Maternal Infection Are Protective for Infant Brain Development. J. Pediatr..

[56] Bahnfleth C., Canfield R., Nevins J., Caudill M., Strupp B. (2019). Prenatal Choline Supplementation Improves Child Color-location Memory Task Performance at 7 Y of Age (FS05-01-19). Curr. Dev. Nutr..

[57] Pereira R.D., De Long N.E., Wang R.C., Yazdi F.T., Holloway A.C., Raha S. (2015). Angiogenesis in the Placenta: The Role of Reactive Oxygen Species Signaling. BioMed Res. Int..

[58] Jiang X., Jones S., Andrew B.Y., Ganti A., Malysheva O.V., Giallourou N., Brannon P.M., Roberson M.S., Caudill M.A. (2014). Choline Inadequacy Impairs Trophoblast Function and Vascularization in Cultured Human Placental Trophoblasts. J. Cell. Physiol..

[59] Kwan S.T.C., King J.H., Yan J., Jiang X., Wei E., Fomin V.G., Roberson M.S., Caudill M.A. (2017). Maternal choline supplementation during murine pregnancy modulates placental markers of inflammation, apoptosis and vascularization in a fetal sex-dependent manner. Placenta.

[60] King J.H., Kwan S.T.C., Yan J., Jiang X., Fomin V.G., Levine S.P., Wei E., Roberson M.S., Caudill M.A. (2019). Maternal Choline Supplementation Modulates Placental Markers of Inflammation, Angiogenesis, and Apoptosis in a Mouse Model of Placental Insufficiency. Nutrients.

[61] Hutzler J.S., Klein H.R., Brenna J.T., Kwan S.T.C., King J.H., Yan J., Wang Z., Jiang X., Roberson M.S., Caudill M.A. (2017). Maternal Choline Supplementation Modulates Placental Nutrient Transport and Metabolism in Late Gestation of Mouse Pregnancy. J. Nutr..

[62] King J.H., Kwan S.T.C., Yan J., Klatt K.C., Jiang X., Roberson M.S., Caudill M.A. (2017). Maternal Choline Supplementation Alters Fetal Growth Patterns in a Mouse Model of Placental Insufficiency. Nutrients.

[63] Nam J., Greenwald E., Jack-Roberts C., Ajeeb T.T., Malysheva O.V., Caudill M.A., Axen K., Saxena A., Semernina E., Nanobashvili K. (2017). Choline prevents fetal overgrowth and normalizes placental fatty acid and glucose metabolism in a mouse model of maternal obesity. J. Nutr. Biochem..

[64] Jack-Roberts C., Joselit Y., Nanobashvili K., Bretter R., Malysheva O.V., Caudill M.A., Saxena A., Axen K., Gomaa A., Jiang X. (2017). Choline Supplementation Normalizes Fetal Adiposity and Reduces Lipogenic Gene Expression in a Mouse Model of Maternal Obesity. Nutrients.

[65] Nanobashvili K., Jack-Roberts C., Bretter R., Jones N., Axen K., Saxena A., Blain K., Jiang X. (2018). Maternal Choline and Betaine Supplementation Modifies the Placental Response to Hyperglycemia in Mice and Human Trophoblasts. Nutrients.

[66] Jansson N., Rosario F.J., Gaccioli F., Lager S., Jones H.N., Roos S., Jansson T., Powell T.L. (2013). Activation of placental mTOR signaling and amino acid transporters in obese women giving birth to large babies. J. Clin. Endocrinol. Metab..

[67] Rosario F.J., Powell T.L., Jansson T. (2016). Activation of placental insulin and mTOR signaling in a mouse model of maternal obesity associated with fetal overgrowth. Am. J. Physiol. Regul. Integr. Comp. Physiol..

[68] Joselit Y., Nanobashvili K., Jack-Roberts C., Greenwald E., Malysheva O.V., Caudill M.A., Saxena A., Jiang X. (2018). Maternal betaine supplementation affects fetal growth and lipid metabolism of high-fat fed mice in a temporal-specific manner. Nutr. Diabetes.

[69] Carlin J., George R., Reyes T.M. (2013). Methyl Donor Supplementation Blocks the Adverse Effects of Maternal High Fat Diet on Offspring Physiology. PLoS ONE.

[70] McCann J.C., Hudes M., Ames B.N. (2006). An overview of evidence for a causal relationship between dietary availability of choline during development and cognitive function in offspring. Neurosci. Biobehav. Rev..

[71] Meck W.H., Williams C.L. (2003). Metabolic imprinting of choline by its availability during gestation: Implications for memory and attentional processing across the lifespan. Neurosci. Biobehav. Rev..

[72] Blusztajn J.K., Slack B.E., Mellott T.J. (2017). Neuroprotective Actions of Dietary Choline. Nutrients.

[73] Mudd A.T., Getty C.M., Dilger R.N. (2018). Maternal Dietary Choline Status Influences Brain Gray and White Matter Development in Young Pigs. Curr. Dev. Nutr..

[74] Getty C.M., Dilger R.N. (2015). Moderate Perinatal Choline Deficiency Elicits Altered Physiology and Metabolomic Profiles in the Piglet. PLoS ONE.

[75] Mudd A.T., Getty C.M., Sutton B.P., Dilger R.N. (2016). Perinatal choline deficiency delays brain development and alters metabolite concentrations in the young pig. Nutr. Neurosci..

[76] Albright C.D., Tsai A.Y., Friedrich C.B., Mar M.-H., Zeisel S.H. (1999). Choline availability alters embryonic development of the hippocampus and septum in the rat. Dev. Brain Res..

[77] Trujillo-Gonzalez I., Friday W.B., Munson C.A., Bachleda A., Weiss E.R., Alam N.M., Sha W., Zeisel S.H., Surzenko N. (2019). Low availability of choline in utero disrupts development and function of the retina. FASEB J..

[78] Gould R.M., Dawson R.M. (1976). Incorporation of newly formed lecithin into peripheral nerve myelin. J. Cell Biol..

[79] Cermak J.M., Holler T., Jackson D.A., Blusztajn J.K. (1998). Prenatal availability of choline modifies development of the hippocampal cholinergic system. FASEB J..

[80] Li Q., Guo-Ross S., Lewis D.V., Turner D., White A.M., Wilson W.A., Swartzwelder H.S. (2004). Dietary Prenatal Choline Supplementation Alters Postnatal Hippocampal Structure and Function. J. Neurophysiol..

[81] Glenn M.J., Gibson E.M., Kirby E.D., Mellott T.J., Blusztajn J.K., Williams C.L. (2007). Prenatal choline availability modulates hippocampal neurogenesis and neurogenic responses to enriching experiences in adult female rats. Eur. J. Neurosci..

[82] Jones J., Meck W.H., Williams C.L., A Wilson W., Swartzwelder H., Iii J.J. (1999). Choline availability to the developing rat fetus alters adult hippocampal long-term potentiation. Dev. Brain Res..

[83] Mellott T.J., Huleatt O.M., Shade B.N., Pender S.M., Liu Y.B., Slack B.E., Blusztajn J.K. (2017). Perinatal Choline Supplementation Reduces Amyloidosis and Increases Choline Acetyltransferase Expression in the Hippocampus of the APPswePS1dE9 Alzheimer’s Disease Model Mice. PLoS ONE.

[84] Velazquez R., Ferreira E., Winslow W., Dave N., Piras I.S., Naymik M., Huentelman M.J., Tran A., Caccamo A., Oddo S. (2019). Maternal choline supplementation ameliorates Alzheimer’s disease pathology by reducing brain homocysteine levels across multiple generations. Mol. Psychiatry.

[85] Thomas J. (2004). Perinatal choline supplementation attenuates behavioral alterations associated with neonatal alcohol exposure in rats. Neurotoxicology Teratol..

[86] Thomas J.D., Abou E.J., Dominguez H.D. (2009). Prenatal choline supplementation mitigates the adverse effects of prenatal alcohol exposure on development in rats. Neurotoxicology Teratol..

[87] Thomas J.D., Tran T.D. (2012). Choline supplementation mitigates trace, but not delay, eyeblink conditioning deficits in rats exposed to alcohol during development. Hippocampus.

[88] Langley E.A., Krykbaeva M., Blusztajn J.K., Mellott T.J. (2015). High maternal choline consumption during pregnancy and nursing alleviates deficits in social interaction and improves anxiety-like behaviors in the BTBR T+Itpr3tf/J mouse model of autism. Behav. Brain Res..

[89] Orenbuch A., Fortis K., Taesuwan S., Yaffe R., Caudill M.A., Golan H.M. (2019). Prenatal Nutritional Intervention Reduces Autistic-Like Behavior Rates Among. Front. Neurosci..

[90] Velázquez R., Ash J.A., Powers B.E., Kelley C.M., Strawderman M., Luscher Z.I., Ginsberg S.D., Mufson E.J., Strupp B.J. (2013). Maternal choline supplementation improves spatial learning and adult hippocampal neurogenesis in the Ts65Dn mouse model of Down syndrome. Neurobiol. Dis..

[91] Powers B.E., Kelley C.M., Velazquez R., Ash J.A., Strawderman M.S., Alldred M.J., Ginsberg S.D., Mufson E.J., Strupp B.J. (2017). Maternal choline supplementation in a mouse model of Down syndrome: Effects on attention and nucleus basalis/substantia innominata neuron morphology in adult offspring. Neuroscience.

[92] Ash J.A., Velázquez R., Kelley C.M., Powers B.E., Ginsberg S.D., Mufson E.J., Strupp B.J. (2014). Maternal choline supplementation improves spatial mapping and increases basal forebrain cholinergic neuron number and size in aged Ts65Dn mice. Neurobiol. Dis..

[93] Yan J., Ginsberg S.D., Powers B., Alldred M.J., Saltzman A., Strupp B.J., Caudill M.A. (2014). Maternal choline supplementation programs greater activity of the phosphatidylethanolamine N-methyltransferase (PEMT) pathway in adult Ts65Dn trisomic mice. FASEB J..

[94] Kennedy B.C., Dimova J.G., Siddappa A.J.M., Tran P.V., Gewirtz J.C., Georgieff M.K. (2014). Prenatal Choline Supplementation Ameliorates the Long-Term Neurobehavioral Effects of Fetal-Neonatal Iron Deficiency in Rats. J. Nutr..

[95] Tran P.V., Kennedy B.C., Pisansky M.T., Won K.-J., Gewirtz J.C., A Simmons R., Georgieff M.K. (2016). Prenatal Choline Supplementation Diminishes Early-Life Iron Deficiency–Induced Reprogramming of Molecular Networks Associated with Behavioral Abnormalities in the Adult Rat Hippocampus. J. Nutr..

[96] Strupp B.J., Powers B.E., Velazquez R., Ash J.A., Kelley C.M., Alldred M.J., Strawderman M., Caudill M.A., Mufson E.J., Ginsberg S.D. (2016). Maternal choline supplementation: A potential prenatal treatment for Down syndrome and Alzheimer’s disease. Curr. Alzheimer Res..

[97] Bekdash R.A., Zhang C., Sarkar D.K. (2013). Gestational choline supplementation normalized fetal alcohol-induced alterations in histone modifications, DNA methylation, and proopiomelanocortin (POMC) gene expression in β-endorphin-producing POMC neurons of the hypothalamus. Alcohol Clin. Exp. Res..

[98] Otero N.K.H., Thomas J.D., Saski C.A., Xia X., Kelly S.J. (2012). Choline supplementation and DNA methylation in the hippocampus and prefrontal cortex of rats exposed to alcohol during development. Alcohol. Clin. Exp. Res..

[99] Resseguie M., Song J., Niculescu M.D., Da Costa K.-A., Randall T.A., Zeisel S.H. (2007). Phosphatidylethanolamine N-methyltransferase (PEMT) gene expression is induced by estrogen in human and mouse primary hepatocytes. FASEB J..

[100] Yan J., Jiang X., West A.A., Perry C.A., Malysheva O.V., Devapatla S., Pressman E., Vermeylen F., Stabler S.P., Allen R.H. (2012). Maternal choline intake modulates maternal and fetal biomarkers of choline metabolism in humans. Am. J. Clin. Nutr..

[101] Schaible T.D., Harris R.A., Dowd S.E., Smith C.W., Kellermayer R. (2011). Maternal methyl-donor supplementation induces prolonged murine offspring colitis susceptibility in association with mucosal epigenetic and microbiomic changes. Hum. Mol. Genet..

[102] Hollingsworth J.W., Maruoka S., Boon K., Garantziotis S., Li Z., Tomfohr J., Bailey N., Potts E.N., Whitehead G., Brass D.M. (2008). In utero supplementation with methyl donors enhances allergic airway disease in mice. J. Clin. Investig..

[103] Giudicelli F., Brabant A.L., Grit I., Parnet P., Amarger V. (2013). Excess of methyl donor in the perinatal period reduces postnatal leptin secretion in rat and interacts with the effect of protein content in diet. PLoS ONE.

[104] Cho C.E., Caudill M.A. (2017). Trimethylamine- N -Oxide: Friend, Foe, or Simply Caught in the Cross-Fire?. Trends Endocrinol. Metab..

[105] Klatt K.C., Caudill M.A. (2017). Pressing the trimethylamine N-oxide narrative. AME Med. J..

